# Intra-Articular Fms-Like Tyrosine Kinase 3 Ligand Expression Is a Driving Force in Induction and Progression of Arthritis

**DOI:** 10.1371/journal.pone.0003633

**Published:** 2008-11-04

**Authors:** Mats Dehlin, Maria Bokarewa, Robert Rottapel, Simon J. Foster, Mattias Magnusson, Leif E. Dahlberg, Andrej Tarkowski

**Affiliations:** 1 Department of Rheumatology and Inflammation Research, Göteborg University, Göteborg, Sweden; 2 Department of Immunology, University of Toronto, Toronto, Ontario, Canada; 3 Department of Molecular Biology & Biotechnology, The University of Sheffield, Sheffield, United Kingdom; 4 Department of Orthopaedics, University Hospital UMAS, Malmo, Sweden; New York University School of Medicine, United States of America

## Abstract

**Background:**

One of the hallmarks of rheumatoid arthritis (RA) is hyperplasia and inflammation of the synovial tissue being characterized by *in situ* occurrence of highly differentiated leukocytes. Fms-like tyrosine kinase 3 (Flt3) has a crucial role in hematopoiesis, regulation of cell proliferation, differentiation and apoptosis. Typically, Flt3 is expressed on early myeloid and lymphoid progenitors and is activated by its soluble ligand (Flt3-L). The highly differentiated cellular pattern in the synovium of the RA patients made us hypothesize that Flt3-L, with its ability to induce proliferation and differentiation, could be of importance in induction and/or progression of arthritis.

**Methodology/Principal Findings:**

To investigate occurrence of Flt3-L in RA we have measured its levels in matched serum and synovial fluid samples from 130 patients and 107 controls. To analyse the pro-inflammatory role of Flt3-L, we continuously overexpressed this protein locally in healthy mouse joints using homologous B-cell line transfected with Flt3-L gene. Additionally, recombinant Flt3-L was instillated intra-articularly in combination with peptidoglycans, a Toll Like Receptor 2-ligand with stong arthritogenic properties. Our results show significantly higher levels of Flt3-L in the synovial fluid as compared to serum levels in RA subjects (p = 0.0001). In addition, RA synovial fluid levels of Flt-3-L were significantly higher than these obtained from synovial fluids originating from non-inflammatory joint diseases (p = 0.022). Intra-articular administration of B-cell line transfected with Flt3-L gene resulted in highly erosive arthritis while inoculation of the same B-cell line without hyperexpression of Flt3-L did not induce erosivity and only in a minority of cases caused synovial proliferation! Flt3-ligand potentiated peptidoglycan induced arthritis as compared to mice injected with peptidoglycan alone (p<0.05).

**Conclusions/Significance:**

Our findings indicate that Flt3-L is strongly expressed at the site of inflammation in human RA. It exerts both pro-inflammatory and tissue destructive properties once in the joint cavity. Owing to these properties, treatment attempts to neutralize this molecule should be considered in RA.

## Introduction

Rheumatoid arthritis (RA) is a chronic, inflammatory, autoimmune joint disease which prognosis has improved over the last decade owing to better pharmacological treatment. However there is only a scarce knowledge regarding pathogenesis of RA. RA gives rise to chronic inflammation and hyperplasia in the joint synovium, development of pannus, and invasion of leukocytes followed by destruction of local articular components such as cartilage and bone. The synovium is normally only a sparsely cellular structure containing adipocytes and scattered blood vessels but in RA the synovium is rich in cells showing a high degree of differentiation, with occurrence of CD4+ T-cells, B-cells, macrophages and dendritic cells. Hyperplasia of the synovium results from a marked increase of macrophage-like and fibroblast-like synoviocytes [1]. The reason for this markedly changed phenotype of joint during inflammation is presently unknown.

The Fms-like tyrosine kinase 3 (Flt3) is a membrane bound tyrosine kinase receptor which has a crucial role in hematopoiesis, regulating cellular differentiation, proliferation and apoptosis. Physiologically, it is mainly expressed on early myeloid and lymphoid progenitors [2] but few studies reported monocyte and granulocyte expression of Flt3 on mRNA and protein levels [3], [4]. The activation of Flt3-mediated signalling is achieved by interaction between Flt3 and its ligand (Flt3-L) leading to dimerization and phosphorylation and resulting in differentiation and proliferation of hematopoietic stem cells, both of myeloid [5] and lymphoid origin [6]. Flt3-L also gives rise to differentiation and clonal expansion of human dendritic cells[7]. Human Flt3-L is a type 1 transmembranous protein consisting of 235 amino acids. The dominating isoform is the full-length transmembrane isoform but there are also soluble forms which consist of different sizes of the extracellular domain. All isoforms are biologically active. In contrast to the Flt3 receptor, the Flt3-L is expressed in most human tissues (spleen, thymus, bone marrow, prostate, kidney, and intestine) but the highest levels are seen in peripheral blood leukocytes. Serum levels of Flt3-L are low in healthy individuals but markedly elevated levels are seen in patients with secondary leukopenia[8].

Flt3-L and its receptor have never been studied in the setting of arthritis. The typical cellular pattern in the synovium of the RA-joint with its abundance of highly differentiated cells implies the possibility that Flt3-L, with its ability to induce differentiation and proliferation, could be of pathogenic importance. To investigate the possible role of Flt3-L in RA we have measured the levels of Flt3-L in serum and synovial fluid of patients and of healthy controls. Furthermore, we wanted to ascertain in vivo role of increased Flt3-L levels in the joint by transplanting Flt3-L secreting cells into healthy mouse joints. Our results show that Flt3-L induces erosive arthritis in mice and that the levels of Flt3-L are significantly elevated in the inflamed joints of RA patients.

## Materials and Methods

### Flt3-L levels in synovial fluids and sera of RA patients

Paired serum (S) and synovial fluid (SF) samples were collected from 130 RA patients who attended the rheumatology clinics at Sahlgrenska University Hospital, Göteborg for acute joint effusion. RA was diagnosed according to the American College of Rheumatology criteria [9]. The patients consisted of 90 females (mean age 61, age range 24–87, mean disease duration 10 years (0–41 years)) and 40 males (mean age 59, age range 25–84, mean disease duration 9 years (0–39 years)). We considered the presence of rheumatoid factor (RF) of any of the major immunoglobulin isotypes as positive. Seventy-eight (51 female, 27 male) of the patients were positive for RF and 52 negative.

Recent radiographs of hand and foot skeleton for all the patients were performed. The presence of bone erosions, defined as the loss of cortical definition in the joint, was recorded in proximal interphalangeal joints, metacarpophalangeal joints, carpus, wrist joints and metatarsophalangeal joints. The presence of one erosion was sufficient to fulfil the requirement of an erosive disease. Seventy-eight (51 female, 27 male) of the patients were erosive and 52 non-erosive. The pharmacotherapy of RA patients is provided in Table 1.

**Table 1:** Clinical data regarding 130 RA-patients and 103 controls participating in the study.

	RA-patients, females (n = 90)	RA-patients, males (n = 40)	Control subjects, serum (n = 70)	Control subjects, synovial fluid (n = 37)
Mean age (SD)	61(17)	59(13)	52(11)	47(21)
Disease duration, years (range)	10 (0–41)	9 (0–39)	–	–
White blood cells, mean (SD)	8.2(2.6)	7.9(2.2)	N.A. *	N.A.
RF positive	51 (57%)	27 (68%)	N.A.	N.A.
Erosive disease	51 (57%)	27 (57%)	N.A.	N.A.
DMARD-treatment	51 (57%)	24 (60%)	None	None
Methotrexate (Mtx) alone	25	12		
Mtx and/or other DMARD	15	8		
Mtx and TNFα-/IL-1-inhibitor	10	4		
Other DMARDs and TNFα-inhibitor	1	0		
No DMARD- treatment	39	16		

* N.A. = not analyzed.

Blood samples from healthy individuals (n = 70; mean age 52 and age range 18–73; 54 females and 16 males) were used as controls. In addition, synovial fluids from patients with traumatic knee joint injuries and osteoarthritis were used as controls (n = 37; mean age 47 and age range 22–88; 17 females and 20 males).

Synovial fluid samples were obtained by arthrocentesis of knee joints. Synovial fluid was aspirated aseptically and transferred into tubes containing sodium citrate (0.129 mol/l; pH 7.4). Simultaneously we obtained blood samples from the cubital vein and directly transferred them into sodium citrate medium. Collected blood and synovial fluid samples were centrifuged at 800×*g* for 15 min, aliquoted and stored frozen at −70°C until use. The study was approved by the Ethics Committee of Sahlgrenska University Hospital. All studies were conducted in compliance with the Declaration of Helsinki, and all patients gave written informed consent to participate in the study.

Flt3-L levels were determined by a sandwich ELISA using a pair of matched antibodies (mouse and goat anti-human Flt3-L; R&D Systems Abingdon, United Kingdom). Briefly, 96-well polystyrene dishes (Nunc, Roskilde, Denmark) were coated with mouse anti-human Flt3-L capture antibodies and incubated over night at 5°C. Following washes with PBS containing 0.05% Tween 20, plates were blocked with 1% bovine serum albumin (BSA, Sigma, St Louis, MO) for one hour. Samples were diluted 1:10 in PBS containing 1% BSA. Biotinylated goat anti-human Flt3-L detection antibodies were used and streptavidin-HRP with corresponding substrate and chromogen were employed for colour development. Double wavelength registration at 450 and 540 nm was used and the difference of absorbances was calculated. The obtained absorbance values were compared with the serial dilutions of recombinant human Flt3-L and are presented as picograms per millilitre.

### Intra-articular injections of Staphylococcus aureus derived peptidoglycan and Flt3-L

We wanted to analyze whether the increased i.a. levels of Flt3-L in RA patients might affect inflammatory responses in vivo. To this end we injected the knee joints of twenty-seven healthy 6-weeks old female NMRI-mice (B&K Universal AB, Stockholm, Sweden) with Staphylococcus aureus derived peptidoglycan (Pep), 10 ng/knee, to induce arthritis. Pep has been shown to induce arthritis when injected intraarticularly into murine knee joints in the amount of 20–100 µg/joint [10]. In thirteen of these mice we also injected 2 ng/knee of recombinant mouse Flt3-L (R&D Systems) together with the peptidoglycan. In control experiments recombinant mouse Flt3-L (R&D Systems) was injected i.a. in the knee joints of healthy 6-weeks old female NMRI-mice at 0.02 ng/joint (n = 5), 0.2 ng/joint (n = 5), and 2 ng/joint (n = 5).

All mice were killed after three days and the knee joints were decalcified in 10% formic acid for 24 hours prior to fixation and paraffin embedding. Serial sections were cut through the whole knee joint and stained with hematoxylin and eosin. Arthritis was evaluated on the scale ranging 0–3 where 0 is normal and 3 severe arthritis. Signs of cartilage and bone erosivity were evaluated separately. All animal experiments were approved by the Ethics Committee of Göteborg University.

### Intra-articular transfer of Flt3-L secreting cells

The mouse hybridoma cell line Sp2.0 transfected with gene for soluble Flt3-L and identical non transfected clone were grown in Dulbecco's modified Eagle's medium (4 mM L-glutamine adjusted to contain 1.5 g/L sodium bicarbonate and 4,5 g/L glucose, 90%; 10% fetal bovine serum and Gentamycin). They were cultured at 37°C, 5% CO2 and passaged twice a week. Immediately prior to injection the cells were centrifuged at 1000 RPM for five minutes and the pellet was resuspended and diluted to proper concentration in sterile PBS.

To ensure that the myeloma cell line was producing Flt3-L, levels of Flt3-L in the cell culture supernatants were determined by a sandwich ELISA using a pair of matched antibodies (R&D Systems). Flt3-L gene transfected cells showed high amount of Flt3-L in supernatant while control myeloma cells did not produce any (data not shown).

In preliminary experiments we injected healthy 6-weeks old female Balb/C mice (B&K Universal AB, Stockholm, Sweden) i.a. in knee joint or intra-peritoneally with the Balb/C derived myeloma cell line transfected with the gene for mouse Flt3-L (Bc/Flt3l+) or with identical cells (Bc/Flt3l-) from the same cell line as a control, respectively. Twelve mice were injected i.p. with 1×10^7^ cells of the myeloma cell line. Six of the mice received the Bc/Flt3l+ cells and six mice received the (Bc/Flt3l-) cells. Half of the mice were killed after three days and the other half after seven days. Intra-peritoneal lavage was performed on all mice with cell counts of the peritoneal fluid. Knee joints were prepared for histological examination.

Next, fifty-eight healthy 6-weeks old female Balb/C mice were injected i.a. in the knee joint with the cell line. The right joint received the (Bc/Flt3l+) and the left joint the (Bc/Flt3l-) cells. The cell numbers were the following: 1×10^6^ cells n = 6, three mice killed after 3 days and three after 7 days; 1×10^5^ cells n = 6, three mice killed after 3 days and three after 7 days; 1×10^4^ n = 36, thirteen mice killed after 3 days, thirteen after 7 days and ten after 30 days; 1×10^3^ cells n = 10, five mice killed after 3 days and five mice after 7 days. After the mice were killed the knee joints were decalcified, fixed and paraffin embedded. Serial sections were cut through the whole knee joint and were stained with hematoxylin and eosin. Degree of arthritis was scored as described above. All animal experiments were approved by the Ethics Committee of Göteborg University.

### Statistical evaluation

The Flt3-L levels in serum and synovial fluid from RA-patients and controls were compared using the Mann-Whitney U-test. We stratified the patient material according to gender, presence of RF, radiological findings (erosive vs non-erosive RA) and calculated the difference regarding Flt3-L levels between the groups using the Mann-Whitney U-test. The relationship between the Flt3-L levels of the RA-patients and duration of disease, age, white blood cell count in serum and synovial fluid, CRP and ESR was calculated employing the Spearman correlation coefficient. The RA-patients were further divided into two cohorts of age, a young (<53 years) and an old (>53 years), and the levels of Flt3-L were compared using the Mann-Whitney U-test.

χ-square-test was performed to evaluate differences on the frequency of arthritis in the mice that received Flt3-L and/or Pep and the mice that received the cell line overexpressing Flt3-L. For the statistical evaluation of the results, *p*<0.05 was considered significant.

## Results

### Flt3-L levels in synovial fluids and sera of RA patients

**Figure 1:**
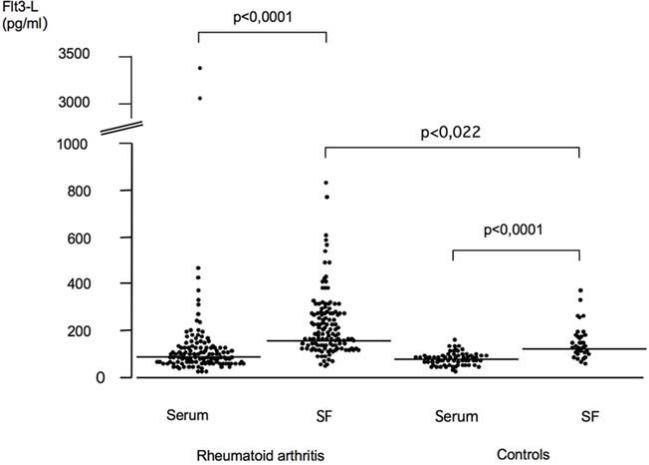
Flt3-L levels in synovial fluids and sera of RA patients and of control subjects. Median displayed in the horizontal line. RA serum (n = 130, median 80, range (min 0–max 3320), RA SF (n = 130, median 160 pg/ml, range (min 20–max 1980), control subjects serum (n = 70, median 70.5 pg/ml, range (min 0–max 160), and control subjects SF (n = 37, median 120 pg/ml, range (min 0–max 360).

We found significantly higher levels of Flt3-L in the synovial fluid (mean 218 pg/ml, SEM 19) as compared to serum levels (mean 141 pg/ml, SEM 35) in RA patients (p = 0.0001) (Fig. 1). In addition, RA synovial fluid levels of Flt-3-L were significantly higher than these obtained from synovial fluids originating from non-inflammatory joint diseases (mean 132 pg/ml, SEM 12) (p = 0.022). There was no significant difference in the circulating Flt3-L levels between patients and controls (mean 74 pg/ml, SEM 4). Interestingly, controls with degenerative/traumatic joint diseases also showed significantly elevated levels of Flt3-L in SF compared to serum (p = 0.0001). Further statistical analysis showed that Flt3-L had a positive correlation to age, both regarding serum and synovial fluid levels. This was not seen in the controls. The RF+ patients displayed significantly increased (p<0.0001) levels of Flt3-L in serum but not in SF compared to RF- patients. The older cohort of RA-patients (>53 years, n = 97) had significantly higher levels of Flt3-L both in serum (p = 0.0155) and SF (p = 0.0232) compared to the younger cohort (<53 years, n = 33).

### Intra-articular co-injection of Staphylococcus aureus derived peptidoglycan and Flt3-L leads to aggravation of arthritis

The histological findings of arthritis in the Pep-injected NMRI-mice were of moderate severity. Microscopic examination of the knee joints showed significantly higher (p<0.05) frequency of arthritis of the mice co-injected with Pep and Flt3-L. Indeed 12 of 13 (92%) mice showed signs of mild to moderate arthritis compared to 8 of 14 (57%) in the group that only received Pep. These data indicate that Flt3-L potentiates peptidoglycan triggered joint inflammation. Notably, a single i.a. injection of Flt3-L (0.02 ng, 0.2 ng, 2 ng/joint) did noy cause joint inflammation.

### Intraarticular transfer of cells overexpressing Flt3-L leads to destructive arthritis

**Figure 2:**
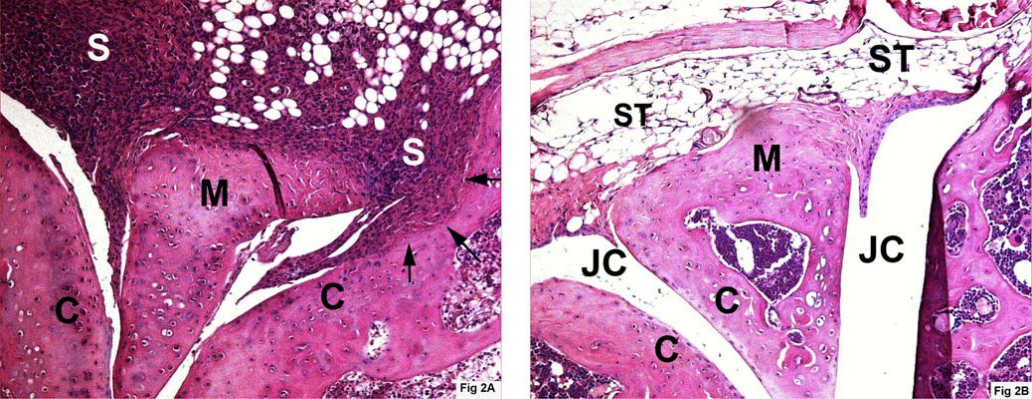
A. Balb/C mouse knee joint 30 days following intra-articular transfer of cells overexpressing Flt3-L. Arrows indicate bone erosions. Abbreviations: C = cartilage, M = meniscus, S = synovitis. B. Balb/C mouse knee joint 30 days following intra-articular transfer of cells not overexpressing Flt3-L. Abbreviations: C = cartilage, JC = joint cavity, M = meniscus, ST = synovial tissue.

Balb/C mice that received i.a. 1×10^4^ Balb/C derived B cell clone transfected with the gene for murine Flt3-L developed after three days histopathological signs of arthritis. Indeed, there was a significantly higher frequency of arthritis since 8/13 (62%) mice developed joint inflammation compared to 3/13 (23%) (p<0.05). After seven days most of the arthritic process has disappeared since only 2/13 mice in each group showed signs of arthritis. The mice that were killed thirty days following injection showed no difference between the groups in frequency of arthritis but there were signs of bone erosions only in the joints that received Flt3-L expressing cells. Five out of ten of the knee joints injected with Flt3-L-expressing cells showed severe signs of arthritis, and all these joints also showed great tumour masses intra- and extraarticularly. Three out of five joints in this group also showed severe destruction of bone (Figure 2). In the control group 4/10 showed signs of arthritis but great tumor masses were only seen in one of the controls and no signs of bone erosions were visible. There were no signs of arthritis when the number of hybridoma cells exceeded 1×10^5^/knee or if the cells were provided i.p.

## Discussion

Our results show that continuous exposure in vivo to Flt3-L induces arthritis in healthy mice. Further, long term exposure to Flt3-L gives rise to significant articular erosivity. We also show that addition of Flt3-L worsens peptidoglycan-induced arthritis in mouse and finally that the levels of Flt3-L are significantly elevated in the synovial fluid of patients with RA.

Flt3-L and its receptor have never been studied in the setting of autoimmune diseases in general or experimental arthritis in particular. We have demonstrated that Flt3-L is strongly expressed at the site of inflammation in human RA. In our material, two known prognostically negative outcome variables for RA, RF+ and high age, correlated to high levels of Flt3-L. Having in mind the arthritogenic properties of Flt3-L, this supports the hypothesis of Flt3-L having a role in induction and/or progression of arthritis. However, in our clinical material we found no correlation to erosive disease. One possible explanation to this is the patient material displaying long disease duration and a large majority of the patients exhibiting established arthritis. In these patients the erosive course of disease has taken place long time ago and the current levels of Flt3-L reflected merely maintenance of arthritis rather than its induction. This calls for further studies of patients with an early disease onset. Indeed, our preliminary data support the notion that in early RA levels of Flt3-L display significant correlation to the erosive course of the disease (Dehlin et al, unpublished).

Three possible mechanisms of action for Flt3-L may contribute to the development of arthritis. Flt3L could increase antigen presentation through increased differentiation of macrophages and dendritic cells, both in the joint but also extra-articularly with dendritic cells migrating to lymph nodes. A second mode of action would be expansion and differentiation of B- and T-cells. This would mainly take place extra-articularly since Flt3-L increases progenitor cells in peripheral blood but these could migrate into the joint. The third, and the most likely explanation, would be Flt3-ligand-mediated activation of synovial dendritic cells, macrophages and fibroblasts giving rise to production of pro-inflammatory cytokines which might initiate joint inflammation even in the absence of T- and B-cells.

The prognosis of RA has lately greatly improved owing to better pharmacologic treatment but this has generated a need for better predictive markers in general and for erosive disease in particular. Our experimental results indicate that Flt3-L could be involved in the erosive process during joint inflammation. Joint destruction is mainly mediated by macrophages and fibroblasts that invade cartilage and activated osteoclasts which in turn cause bone resorption. Osteoclasts are derived from mononuclear phagocyte precursors in the presence of RANKL and macrophage colony-stimulating factor (M-CSF). A recent study has shown that Flt3-L can substitute M-CSF in support of osteoclast differentiation and function [11]. Flt3-L has also been shown to mobilize osteoclastogenic progenitors in non-human primates in vivo [12]. Thus, the elevated levels of Flt3-L in the synovial fluid might indeed stimulate osteoclast differentiation and thereby become a mediator of erosive disease.

In this study we provide support for Flt3-L concerning both development of and erosive course of arthritis. These data will be further validated in a large prospective RA study presently being evaluated.
